# Assessing the predictive potential of ADAM8 for disease control in chronic rhinosinusitis with nasal polyps

**DOI:** 10.3389/falgy.2024.1488441

**Published:** 2024-11-11

**Authors:** Peiqiang Liu, Meng Liu, Yibin Sun, Weiwei Lei, Yu Xu

**Affiliations:** ^1^ Department of Otolaryngology-Head and Neck Surgery, Renmin Hospital of Wuhan University, Wuhan, China; ^2^ Department of Rhinology and Allergy, Renmin Hospital of Wuhan University, Wuhan, China; ^3^ Hubei Province Key Laboratory of Allergy and Immunology, Wuhan, China

**Keywords:** chronic rhinosinusitis with nasal polyp, ADAM8, biomarker, endotypes, disease control status

## Abstract

**Background:**

A disintegrin and metalloproteinase 8 (ADAM8) has been implicated in eosinophilic inflammation; however, its role in chronic rhinosinusitis with nasal polyps (CRSwNP) remains to be elucidated. This study aimed to investigate the predictive significance of ADAM8 levels in nasal secretions for the endotypes and disease control status of CRSwNP.

**Methods:**

A cohort comprising 120 CRSwNP patients and 45 healthy controls (HCs) was assembled, delineating 53 non-eosinophilic CRSwNP (neCRSwNP) and 67 eosinophilic CRSwNP (eCRSwNP) patients. Immunohistochemistry and enzyme-linked immunosorbent assay (ELISA) were utilized to measure ADAM8 levels in nasal mucosal tissues and secretions from all participants. The receiver operating characteristic (ROC) curves and Pearson correlation analysis were employed to assess the predictive capability of ADAM8 levels in predictiving CRSwNP endotypes and disease control status.

**Results:**

ADAM8 levels in nasal secretions were elevated in CRSwNP patients compared to HCs, with a more pronounced increase observed in eCRSwNP patients. Elevated ADAM8 concentrations in nasal secretions were positively correlated with peripheral blood eosinophil counts and percentages, tissue eosinophil counts, serum total IgE, Lund–Mackay scores, and Lund-Kennedy scores. Ultimately, 103 CRSwNP patients completed the follow-up protocol, with 72 classified as the controlled group and 31 as the uncontrolled group. Uncontrolled CRSwNP patients exhibited significantly higher ADAM8 levels in nasal secretions compared to the controlled group. The ROC curves indicated that ADAM8 in nasal secretions exhibits robust discriminatory capacity for eCRSwNP and postoperative disease control status.

**Conclusion:**

ADAM8 in nasal secretions emerges as a potential novel biomarker for the prognostication of CRSwNP endotypes and the postoperative disease control status.

## Introduction

Chronic rhinosinusitis with nasal polyps (CRSwNP) is characterized by a significant degree of heterogeneity within its patient population (1). The disease is clinically stratified into eosinophilic CRSwNP (eCRSwNP) and non-eosinophilic CRSwNP (neCRSwNP), based on the degree of tissue eosinophilic infiltration (2, 3). The advent of diverse therapeutic options has facilitated a more nuanced therapeutic approach tailored to each endotype (4–6). However, existing classification methodologies are mainly based on postoperative histopathology. Consequently, the preoperative differentiation of eCRSwNP and neCRSwNP to facilitate targeted therapeutic strategies and enhance patient prognoses presents an area ripe for further investigation.

A disintegrin and metalloproteinase 8 (ADAM8), belonging to the “a disintegrin and metalloproteinase” family and identified by the leukocyte differentiation antigen CD156 (7–9). It is present in the body in both intracellular and extracellular secreted forms. In the respiratory tract, ADAM8 is predominantly localized on inflammatory cells encircling bronchi and blood vessels, as well as on epithelial cells. Notably, its expression is heightened on eosinophils, mononuclear macrophages, and neutrophils that have infiltrated the tissue (10–12). Studies have reported a significant increase in soluble ADAM8 levels in the sputum of asthmatic patients, correlating inversely with the forced expiratory volume in one second (FEV1), a measure of pulmonary function (13, 14). In eosinophilic pneumonia, soluble ADAM8 levels in bronchoalveolar lavage fluid (BALF) and peripheral blood are also found to be notably elevated relative to control groups (15, 16). Despite the established role of ADAM8 in various respiratory conditions, its specific function within the nasal secretions of patients with CRSwNP remains to be elucidated. To bridge this knowledge gap, a single-center, prospective study has been initiated with the aim of evaluating the clinical relevance of ADAM8 levels in nasal secretions for the preoperative differentiation of CRSwNP endotypes and the prediction of disease control status.

## Materials and methods

### Participants and settings

This investigation enrolled a cohort of 120 patients diagnosed with CRSwNP and a control group of 45 healthy controls (HCs), all of whom underwent endoscopic surgical procedures at the Renmin Hospital of Wuhan University between January 2023 and June 2023. The inclusion criteria for CRSwNP patients were as follows: (1) The diagnosis conformed to the guidelines set forth by the European Position Paper on Rhinosinusitis and Nasal Polyps 2020 (EPOS 2020) (17); (2) Participants were over the age of 18 years; (3) No prior use of immunomodulatory agents or glucocorticoids within a four-week period preceding surgery. Exclusion criteria for CRSwNP patients included: (1) Individuals with nasal and sinus tumors, fungal sinusitis, and other structural anomalies of the nasal and sinus cavities, as well as those with immune system disorders leading to nasal and sinus masses; (2) A history of autoimmune diseases and metabolic disorders. The HC group consisted of only a deviated nasal septum and no clinical manifestations of rhinitis, sinusitis, or any inflammatory or autoimmune disease. The study protocol was granted approval by the Medical Ethics Committee of our institution (No. WDRY2022-K227), and informed consent was obtained from all participants prior to surgical intervention.

### EPOS disease control assessment

The primary endpoints were time-to-event measures, focusing on the post-surgical control of CRSwNP at one year. Adhering to the EPOS 2020 guidelines (17), the study delineated seven pivotal metrics for evaluating CRSwNP control, including the quantification of five cardinal symptoms’ severity over the preceding month—nasal congestion, rhinorrhea or postnasal drip, facial pain or pressure, olfactory dysfunction, and sleep disturbance or fatigue—as well as the consumption of rescue medications within the preceding six months and the detection of pathological mucosa via nasal endoscopy. Patient-reported symptom severity was measured using a VAS score, scaled from 0 to 10. A VAS score ≤5 suggests minimal or no impact on the patient's quality of life, whereas a score >5 indicates symptomatic distress throughout the majority of the week. The classification of CRSwNP control status adhered to the EPOS guidelines and previous studies (17–19): “controlled” if none of the seven criteria were met, and “uncontrolled” with three or more criteria met.

### Endotypes of CRSwNP

The nasal polyp tissue underwent histological examination through hematoxylin-eosin (HE) staining. A systematic sampling approach was implemented, wherein five high-power fields (HPF, at a magnification of ×400) were randomly selected. The mean count of eosinophils was then determined from these fields. The diagnosis of eCRSwNP is established when the tissue eosinophil proportion reaches or exceeds a threshold of 10%. Conversely, a tissue eosinophil proportion of less than 10% is indicative of neCRSwNP (3, 4).

### Nasal secretion collection and ADAM8 level detection

A sterile expandable sponge was meticulously inserted into the patient's nasal cavity, remaining there for a period of 30 min prior to its careful extraction. The secretions were expressed by applying pressure with a syringe. The collected material was then transferred into a centrifuge tube and stored at −80 ℃ for subsequent analysis. For the quantification of ADAM8 in the nasal secretions, a specific enzyme-linked immunosorbent assay (ELISA) kit was employed, sourced from Shanghai Enzyme-Linked Biotechnology Co., Ltd. The protocol was executed with rigorous adherence to the manufacturer's guidelines.

### Nasal nitric oxide measurement

The assessment of nasal fractional exhaled nitric oxide (FnNO) levels was conducted using a nitric oxide (NO) analyzer manufactured by Sunvou Corporation in Wuxi, China. Participants were positioned in a seated posture, with an olive-shaped nasal occluder inserted into one nostril. Subsequently, subjects were instructed to maintain a slight closure of their mouth and to engage in normal respiratory activities. The apparatus continuously extracted exhaled gases through a sampling tube, maintaining a flow rate of 5 milliliters per second. To ensure the precision of the measurements, the protocol stipulated the execution of a minimum of three trials, with the resultant FnNO values being averaged.

### Immunohistochemistry

Nasal polyp tissues were fixed with a 4% paraformaldehyde solution for a period of 24 h, followed by the preparation of paraffin-embedded sections. The anti-ADAM8 antibody (1:100, Proteintech) was applied and incubated at 4°C overnight. Subsequently, biotinylated goat anti-rabbit IgG was added and incubated for 30 min. The chromogenic reaction was then performed using 3,3'-diaminobenzidine (DAB). The absorbance was measured using the Image-Pro Plus 6.0 system.

### Statistical analysis

All data were expressed as mean ± standard deviation. When the variables were normally distributed, one-way analysis of variance (ANOVA) or Student's *t*-test was performed; otherwise, the Kruskal–Wallis H test or Mann–Whitney *U* test was utilized. Categorical data were presented as numbers (%), and comparisons were made using the chi-square test. The correlation between ADAM8 expression and clinical variables was analyzed using the Pearson correlation analysis. The predictive value of ADAM8 levels for eCRSwNP and postoperative disease control status was evaluated through the receiver operating characteristic (ROC) curve. A *P* value of less than 0.05 was considered to be statistically significant.

## Results

### Demographic and clinical features between eCRSwNP and neCRSwNP patients

**Figure 1:**
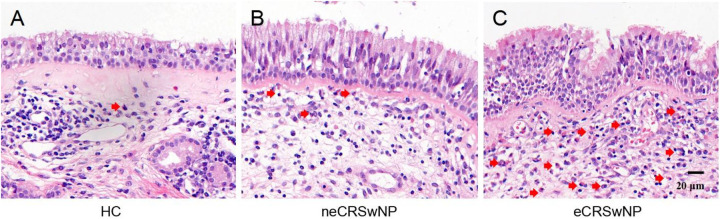
Pepresentative HE-stained images of HC **(A)**, neCRSwNP **(B)** and eCRSwNP **(C)** groups. Eosinophils are marked by red arrows.

Demographic and clinical data of all participants are presented in Table 1. Representative HE-stained images of HC, neCRSwNP and eCRSwNP groups were shown in Figure 1. No significant differences were observed among the three groups in terms of gender, age, asthma, smoking, peripheral blood eosinophil percentage, serum total IgE levels, FnNO, VAS scores, Lund-Mackay scores, and Lund-Kennedy scores (*P* > 0.05). However, the prevalence of allergic rhinitis and peripheral blood eosinophil count in the eCRSwNP group were significantly higher compared to the other two groups (*P* < 0.05).

**Table 1:** The demographic and clinical features of participants among three groups.

Variables	HC	neCRSwNP	eCRSwNP	*P*
Number	45	53	67	–
Age (years)	38.16 ± 2.01	40.85 ± 2.19	41.60 ± 1.86	0.478
Sex (M/F)	25/20	29/24	31/36	0.535
Smoking (Y/N)	9/36	11/42	13/54	0.983
Allergic rhinitis (Y/N)	0/45	13/40	16/51	0.001
Asthma (Y/N)	0/45	5/48	8/59	0.062
Blood eosinophil counts (10^9^/L)	0.30 ± 0.05	0.32 ± 0.04	0.46 ± 0.04	0.014
Blood eosinophil percentage (%)	3.63 ± 0.59	3.88 ± 0.39	3.35 ± 0.36	0.660
Tissue eosinophil counts (n/HPF)	–	4.74 ± 0.36	20.27 ± 1.20	<0.001
Tissue eosinophil percentage (%/HPF)	–	5.24 ± 1.33	17.04 ± 3.43	<0.001
Serum total IgE (IU/ml)	155.25 ± 35.86	265.21 ± 45.21	286.57 ± 37.69	0.063
FnNO (ppb)	220.76 ± 22.94	219.79 ± 21.93	207.60 ± 19.61	0.884
VAS score	–	4.34 ± 0.32	3.96 ± 0.30	0.704
Lund–Mackay score	–	11.17 ± 0.73	10.78 ± 0.58	0.350
Lund–Kennedy score	–	5.04 ± 0.44	6.61 ± 0.41	0.752

HC, healthy control; neCRSwNP, non-eosinophilic chronic rhinosinusitis with nasal polyps; eCRSwNP, eosinophilic chronic rhinosinusitis with nasal polyps; HPF, high power field; FnNO, nasal fractional exhaled nitric oxide; VAS, visual analogue scale.

### ADAM8 levels in nasal secretion and tissues of CRSwNP

**Figure 2:**
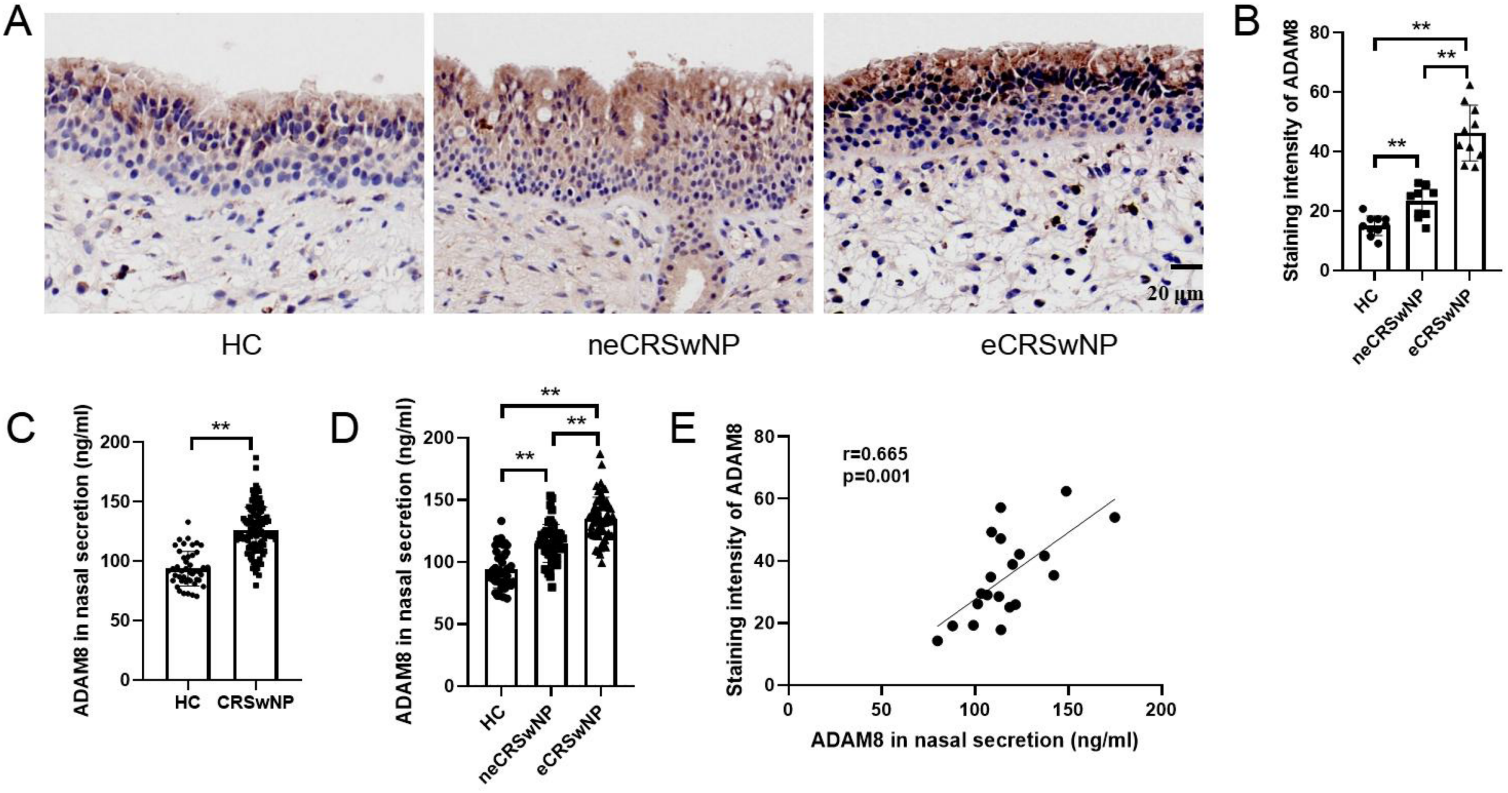
ADAM8 levels in nasal secretion and tissues of CRSwNP. **(A,B)** The staining intensity of ADAM8 were detected by immunohistochemistry. *n* = 10 for each group. **(C)** ADAM8 concentrations in nasal secretions in the HC and CRSwNP groups. *n* = 45 for HC group, *n* = 120 for CRSwNP group. **(D)** ADAM8 concentrations in nasal secretions in the HC and CRSwNP groups. *n* = 45 for HC group, *n* = 53 for neCRSwNP group, *n* = 67 for neCRSwNP group. **(E)** Pearson correlation analysis was used to analyze the correlation between ADAM8 in nasal secretions and in tissues. *n* = 20 for CRSwNP group. The multiple groups were analyzed by one-way ANOVA followed by *post hoc* Tukey's tests for multiple comparisons, while an unpaired Student t test was utilized for binary comparisons. *P < 0.05, **P < 0.01.

In comparison to the HC group, both the eCRSwNP and neCRSwNP groups exhibited significantly elevated levels of ADAM8 in nasal polyp tissues, with eCRSwNP demonstrating higher levels than neCRSwNP (Figures 2A,B). Correspondingly, when contrasted with the HC group, the CRSwNP group showed a statistically significant increase in ADAM8 levels within nasal secretions (*P* < 0.05). Notably, the eCRSwNP group presented with significantly higher ADAM8 levels in nasal secretions compared to the neCRSwNP group (*P* < 0.05) (Figures 2C,D). The Pearson correlation analysis indicated a robust correlation between the ADAM8 levels in nasal secretions and those in the corresponding tissue samples (Figure 2E).

### Evaluation of the predictive value of ADAM8 for CRSwNP endotypes

**Figure 3:**
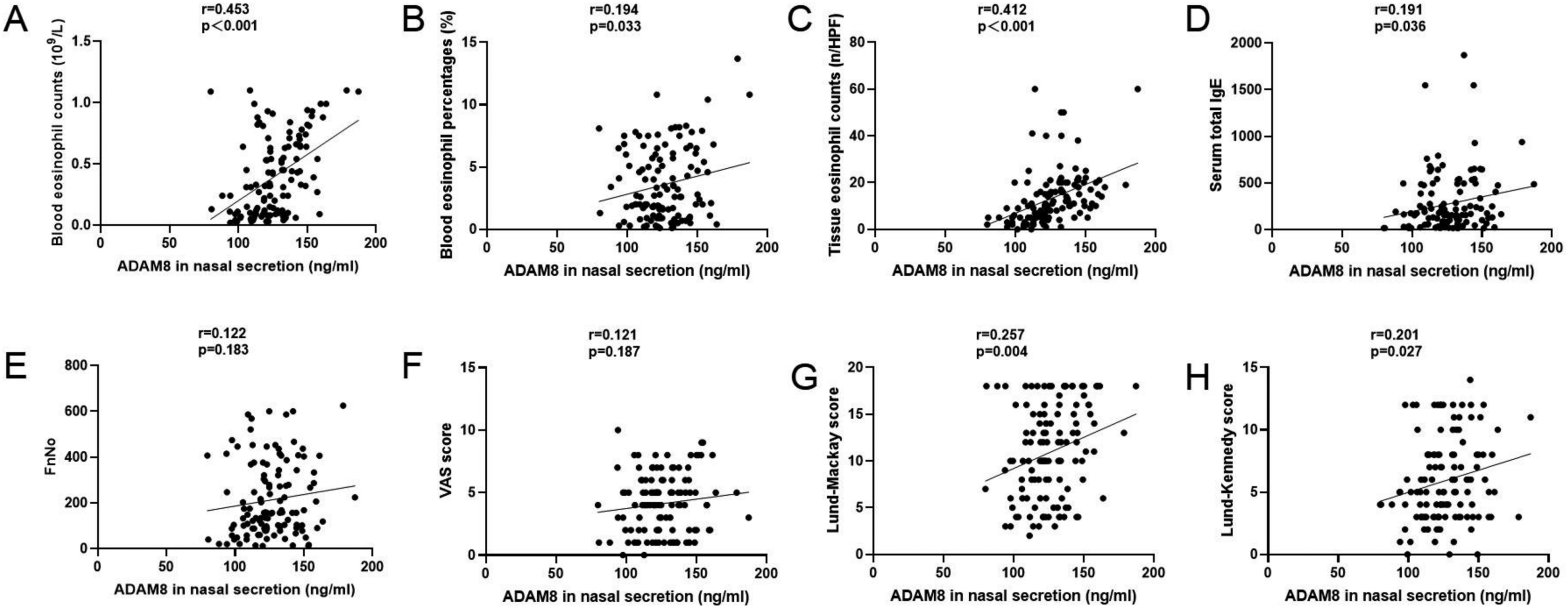
ADAM8 levels in CRSwNP patients and the associations with clinical variables. Pearson correlation analysis results exhibited that ADAM8 concentrations associated with blood eosinophil counts **(A)** and percentages **(B)**, tissue eosinophil counts **(C)**, serum total IgE **(D)**, FnNO **(E)**, VAS scores **(F)**, Lund–Mackay scores **(G)**, and Lund-Kennedy scores **(H)** *n* = 120 for CRSwNP group. *P < 0.05, **P < 0.01.

**Figure 4:**
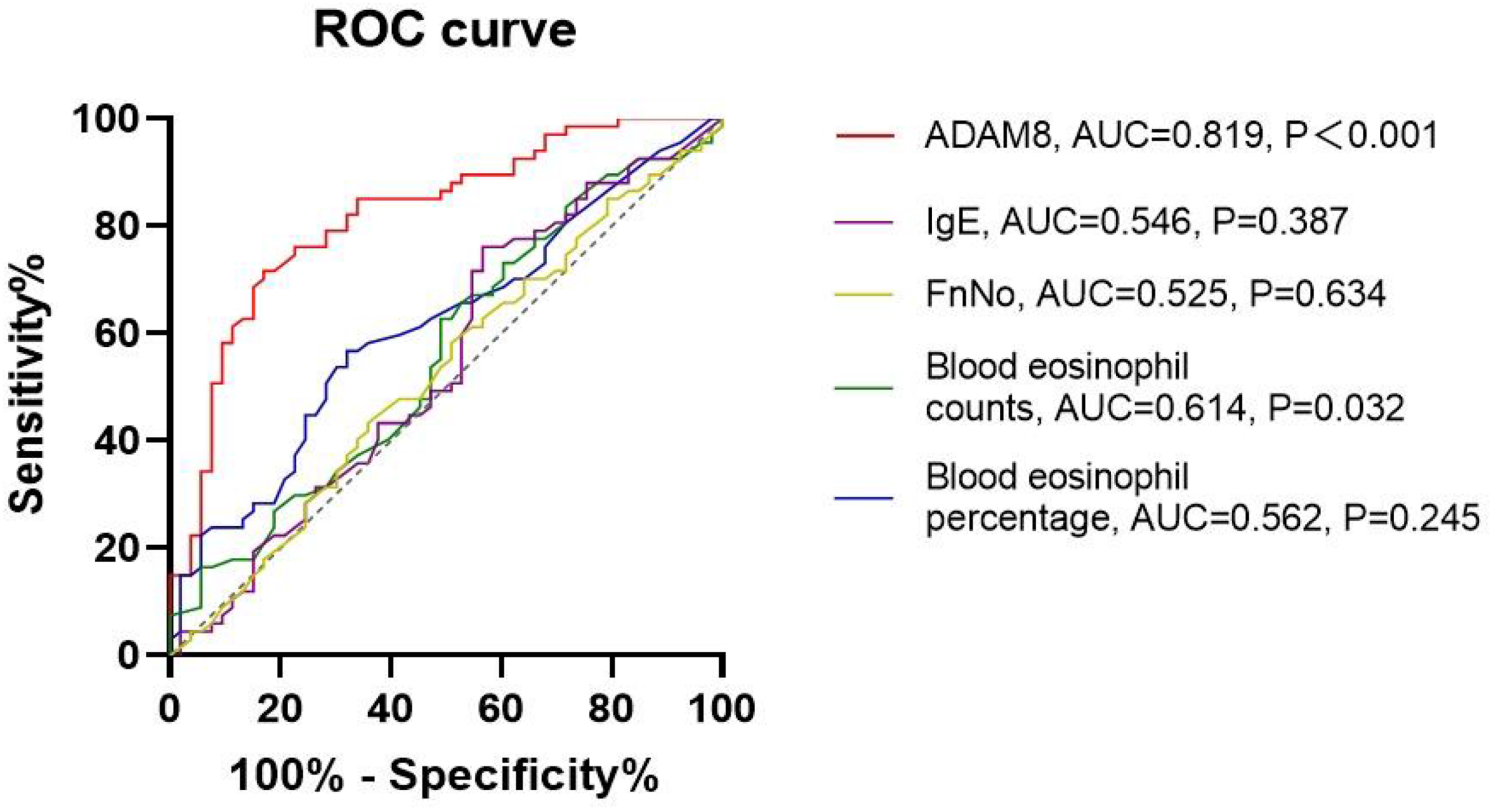
ROC curves of potential predictive variables for distinguishing CRSwNP endotypes. ADAM8 level showed better accuracy and reliability for distinguishing eCRSwNP (AUC = 0.819) than blood eosinophil counts (AUC = 0.614), blood eosinophil percentages (AUC = 0.562), FnNO (AUC = 0.525) and serum total IgE (AUC = 0.546). *P < 0.05, **P < 0.01.

Elevated levels of ADAM8 in nasal secretions were significantly correlated with peripheral blood eosinophil count (*r* = 0.453, *P* < 0.001) and percentage (*r* = 0.194, *P* = 0.033), tissue eosinophil count (*r* = 0.412, *P* < 0.001), serum total IgE levels (*r* = 0.191, *P* = 0.036), Lund-Mackay scores (*r* = 0.257, *P* = 0.004), and Lund-Kennedy scores (*r* = 0.201, *P* = 0.027). No significant correlation was observed with FnNO or VAS scores (*P* > 0.05) (Figure 3). The ROC curve analysis revealed that the predictive accuracy of ADAM8 levels in nasal secretions, with an area under the curve (AUC) of 0.819 (*P* < 0.001), was superior to that of peripheral blood eosinophil count (AUC = 0.614, *P* = 0.032), blood eosinophil percentage (AUC = 0.562, *P* = 0.245), serum total IgE levels (AUC = 0.546, *P* = 0.387), and FnNO (AUC = 0.525, *P* = 0.634) for the CRSwNP endotypes (Figure 4).

### Demographic and clinical features between controlled and uncontrolled CRSwNP patients

Ultimately, a total of 103 patients with CRSwNP completed the postoperative follow-up protocol, with 72 classified into the controlled group and 31 into the uncontrolled group. Demographic and clinical data for all participants are presented in Table 2. No significant differences were observed between the two groups in terms of gender, age, allergic rhinitis, asthma, smoking, peripheral blood eosinophil percentage, VAS scores, and FnNO levels (*P* > 0.05). However, the uncontrolled CRSwNP group exhibited significantly higher peripheral blood eosinophil counts, serum total IgE levels, Lund-Mackay scores, and Lund-Kennedy scores compared to the controlled group (*P* < 0.05).

**Table 2:** The demographic and clinical features between controlled and uncontrolled CRSwNP patients.

Variables	Controlled CRSwNP	Uncontrolled CRSwNP	*P*
Number	72	31	–
Age (years)	39.50 ± 2.84	42.01 ± 3.13	0.064
Sex (M/F)	42/30	13/18	0.126
Smoking (Y/N)	17/55	6/25	0.634
Allergic rhinitis (Y/N)	13/59	7/24	0.594
Asthma (Y/N)	5/67	4/27	0.326
Blood eosinophil counts (10^9^/L)	0.29 ± 0.04	0.50 ± 0.06	0.002
Blood eosinophil percentage (%)	3.26 ± 0.31	4.38 ± 0.85	0.136
Tissue eosinophil counts (n/HPF)	12.01 ± 1.47	20.55 ± 1.39	<0.001
Serum total IgE (IU/ml)	165.67 ± 27.71	316.87 ± 54.92	0.007
FnNO (ppb)	201.55 ± 19.28	267.61 ± 28.04	0.059
VAS score	4.01 ± 0.42	5.03 ± 0.46	0.052
Lund–Mackay score	10.19 ± 0.60	12.84 ± 0.76	0.012
Lund–Kennedy score	5.64 ± 0.36	7.65 ± 0.66	0.004

Controlled CRSwNP, controlled chronic rhinosinusitis with nasal polyps; Uncontrolled CRSwNP, uncontrolled chronic rhinosinusitis with nasal polyps; HPF, high power field; FnNO, nasal fractional exhaled nitric oxide; VAS, visual analogue scale.

### Evaluation of the predictive value of ADAM8 for disease control status

**Figure 5:**
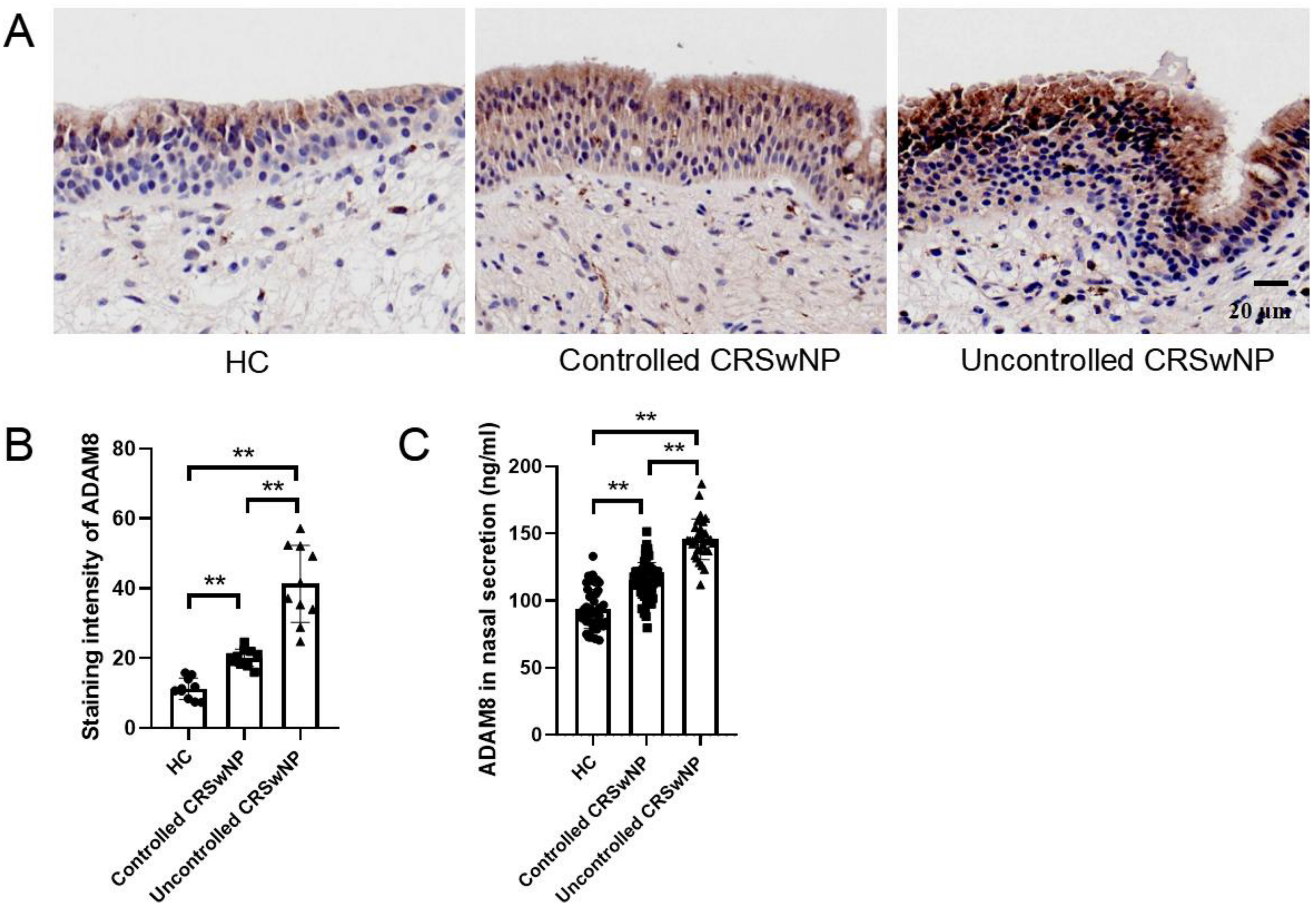
ADAM8 levels in nasal secretion and tissues of controlled and uncontrolled CRSwNP patients. **(A,B)** The staining intensity of ADAM8 were detected by immunohistochemistry. *n* = 10 for each group. **(C)** ADAM8 concentrations in nasal secretions in the HC, controlled CRSwNP and uncontrolled CRSwNP groups. *n* = 45 for HC group, *n* = 72 for controlled CRSwNP group, *n* = 31 for uncontrolled CRSwNP group. The multiple groups were analyzed by one-way ANOVA followed by *post hoc* Tukey's tests for multiple comparisons. *P < 0.05, **P < 0.01.

**Figure 6:**
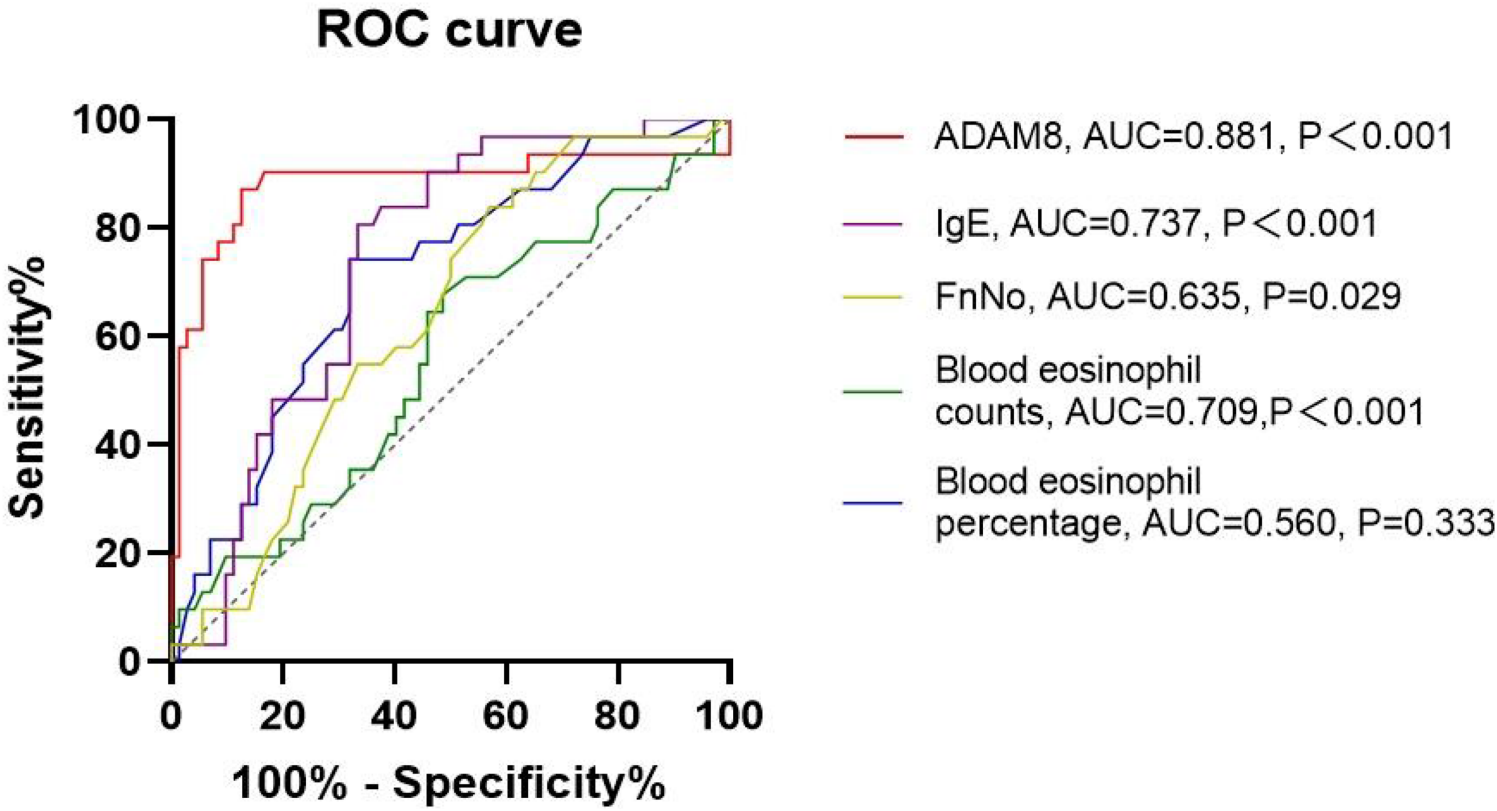
ROC curves of potential predictive variables for distinguishing disease control status. ADAM8 level showed better accuracy and reliability for distinguishing uncontrolled CRSwNP (AUC = 0.881) than blood eosinophil counts (AUC = 0.709), blood eosinophil percentages (AUC = 0.560), FnNO (AUC = 0.635) and serum total IgE (AUC = 0.737). *P < 0.05, **P < 0.01.

In comparison to the HC group, both the uncontrolled and controlled CRSwNP groups exhibited significantly elevated levels of ADAM8 in nasal polyp tissues, with the uncontrolled CRSwNP group demonstrating higher levels than the controlled group. Correspondingly, the increase in ADAM8 levels in nasal secretions was more pronounced in the uncontrolled CRSwNP group (Figure 5). The ROC curve analysis indicated that the predictive accuracy of ADAM8 levels in nasal secretions, with an AUC of 0.881 (*P* < 0.001), was superior for assessing the disease control status of CRSwNP when compared to peripheral blood eosinophil count (AUC = 0.709, *P* < 0.001), blood eosinophil percentage (AUC = 0.560, *P* = 0.333), serum total IgE levels (AUC = 0.737, *P* < 0.001), and FnNO (AUC = 0.635, *P* = 0.029) (Figure 6).

## Disccusion

CRSwNP is stratified into two endotypes based on the degree of eosinophilic infiltration within the tissue: eCRSwNP and neCRSwNP. The eCRSwNP is predominantly characterized by a Th2 inflammatory response, whereas neCRSwNP is associated with Th1, Th3, or mixed inflammatory patterns (6). Patients with eCRSwNP typically present with more severe clinical symptoms and a higher comorbidity rate of asthma (20). The eCRSwNP shows a better response to glucocorticoid therapy, whereas neCRSwNP exhibits greater sensitivity to macrolide antibiotics (21). Furthermore, eCRSwNP is associated with a higher recurrence rate and a higher proportion of refractory cases post-surgery (22). Given the high heterogeneity and recurrence rates of this disease, there is an urgent need to identify novel biomarkers that can distinguish CRSwNP endotypes. The examination of biomarkers within nasal secretions emerges as a promising non-invasive diagnostic modality, attributable to its simplicity, patient acceptability, and fidelity in mirroring the localized inflammatory response (23, 24). In this study, we observed elevated levels of ADAM8 in the nasal secretions of CRSwNP patients, which correlated with eosinophil counts and percentages in tissue and serum, as well as serum total IgE levels. Moreover, the ROC curve analysis indicates that ADAM8 possesses potential accuracy and reliability in discerning CRSwNP endotypes.

Investigations have revealed that ADAM8 is not only implicated in the pathogenesis of asthma but also closely associated with the severity of asthma and indices of pulmonary function (8, 9, 13, 15, 25). In individuals with asthma, ADAM8 is predominantly expressed within eosinophils in sputum samples, and its expression is significantly elevated compared to that of the control group (13, 14). Correlation analysis with pulmonary function tests has demonstrated a marked negative correlation between ADAM8 levels in sputum and the FEV1, an indicator of pulmonary function, suggesting a close relationship between ADAM8 levels in sputum and the severity of asthma (13). Expression of ADAM8 is significantly increased in moderate and severe asthma groups compared to mild and normal groups, with no significant difference observed between the severe and moderate groups (10). Soluble ADAM8 levels are significantly elevated in bronchoalveolar lavage fluid in both acute and chronic eosinophilic pneumonia compared to the control group, and a similar increase is observed in peripheral blood of patients with eosinophilic pneumonia (15, 16). Integrating previous observations with the findings of the present study, we hypothesize that the overproduction of ADAM8 in nasal polyps may facilitate its infiltration into the extracellular space in a soluble form. Ultimately, an increase in local ADAM8 concentration may exacerbate eosinophilic inflammation in polyp tissue.

Investigations have consistently demonstrated that the magnitude of eosinophilic infiltration within nasal polyp tissues is predictive, to a notable degree, of the post-treatment recurrence rate in CRSwNP. Similarly, a direct correlation exists between the peripheral blood eosinophil count and the recurrence rate of CRSwNP (22, 26). In recent years, biologic agents targeting Th2 immune responses, such as Omalizumab (anti-IgE), Mepolizumab (anti-IL-5), and Dupilumab (anti-IL-4 receptor α), have demonstrated effective treatment outcomes in CRSwNP patients (27–30). Guidelines also recommend the use of short-term oral glucocorticoids in conjunction with the aforementioned biologics for the treatment of eCRSwNP (17). In theory, the use of biomarkers to accurately predict the therapeutic prognosis of CRSwNP following novel treatment regimens is of paramount importance for providing personalized and precision medicine to CRSwNP patients. In the present study, we initially observed significant differences in ADAM8 levels between controlled and uncontrolled CRSwNP patients; ROC analysis revealed a close association between ADAM8 levels and poor postoperative disease control. Combined with the above studies, we hypothesize that elevated concentrations of ADAM8 may enhance Th2 immune responses and eosinophil recruitment in polyp tissue, exacerbating eosinophilic inflammation and increasing the risk of adverse prognosis and recurrence. These findings suggest that ADAM8 could be a reliable predictor of postoperative disease control in CRSwNP.

In conclusion, this investigation reveals that among individuals afflicted with CRSwNP, specifically eCRSwNP, there exists an augmented expression of ADAM8 in nasal secretions, which correlates with the severity of eosinophilic infiltration and postoperative disease control status. Given that ADAM8 in nasal secretions can be collected and assayed non-invasively, it holds potential as a novel biomarker for preoperatively distinguishing CRSwNP endotypes and the prognostication of postoperative disease control status.

## Data Availability

The original contributions presented in the study are included in the article/Supplementary Material, further inquiries can be directed to the corresponding author.
